# Neurosurgical management of paediatric central nervous system tumours in low, middle and high-income countries: a multi-centre, international, cross-sectional study

**DOI:** 10.1007/s10143-026-04135-x

**Published:** 2026-01-31

**Authors:** Solange Bramer, Soham Bandyopadhyay, Ruth Mitchell, Andreas K. Demetriades, Ronnie E. Baticulon, Jogi Pattisapu, Andres Rubiano, Nqobile Thango, Kokila Lakhoo, Global Children’s NCDs Collaborative

**Affiliations:** 1 Nuffield Department of Surgical Sciences, Oxford University Global Surgery Group, University of Oxford, Oxford, UK; 2 Clinical Neurosciences, Clinical & Experimental Sciences, Faculty of Medicine, University of Southampton, Southampton, Hampshire, UK; 3 Wessex Neurological Centre, University Hospital Southampton NHS Foundation Trust, Southampton, UK; 4 Department of Neurosurgery, Sydney Children’s Hospital, New South Wales Health, Randwick, NSW Australia; 5 Department of Neurosurgery, Royal Infirmary Edinburgh, Edinburgh, Scotland, UK; 6 Leiden University Medical Centre, Leiden, The Netherlands; 7 Division of Neurosurgery, Department of Neurosciences, Philippine General Hospital, University of the Philippines Manila, Manila, Philippines; 8 College of Medicine, University of Central Florida, Orlando, Fl USA; 9 Neuroscience Institute, Universidad El Bosque, Bogota, Colombia; 10 Division of Neurosurgery, Department of Surgery, University of Cape Town, Cape Town, South Africa; 11 Neuroscience Institute, University of Cape Town, Cape Town, South Africa

**Keywords:** Global surgery, Paediatrics, Global neurosurgery, Neuro-oncology, Paediatric neurosurgery

## Abstract

Paediatric central nervous system (CNS) tumours remain a leading cause of cancer-related mortality worldwide. This may partly be because the distribution of vital diagnostic and therapeutic resources is highly uneven across the world. This study characterised paediatric neuro-oncology services across countries of differing economic and development statuses to identify potential sources of disparities in care. This web-based survey was completed by doctors managing CNS tumours at different healthcare facilities internationally. Questions covered participant demographics, institution type, institutional workforce, imaging, surgical, chemotherapy and radiotherapy availability, and multidisciplinary team (MDT) structures. Responses were categorised by World Bank income level, Human Development Index, and Sustainable Development Index. Chi-square and ANOVA tests were used. The participants were 139 clinicians from 122 healthcare facilities across 110 cities/towns in 49 countries: 29 were LMICs. Of 138 respondents reporting their role, 120 (87.0%) were neurosurgical consultants, with 90 (75.0%) being paediatric neurosurgery specialists. Centres in high-income countries were significantly more likely to have a dedicated paediatric neurosurgeon (97.1%, *p* < 0.001) and MDT services (93.9% vs. 40.0% in low-income settings, *p* < 0.001). Facilities in high income countries were more likely to have neurosurgical trainees (*p* = 0.001). Magnetic resonance imaging was readily available in most high-income centres but less so in low-income centres (*p* < 0.001); positron emission tomography was absent in all low-income settings (*p* < 0.001). Chemotherapy and radiotherapy availability declined with lower income (*p* = 0.007 and *p* = 0.005 respectively). Median one-year follow-up rates were highest in high-income countries (100%) and lowest in low-income settings (34%). These results underscore wide disparities in paediatric neuro-oncology resources, with limited specialist training, imaging technology, and oncology services in lower-income areas. Targeted programmes to bolster local neurosurgical fellowships, telemedicine-based MDT coordination, teleradiology, and sustainable funding for imaging and adjuvant therapies could collectively improve care and follow-up rates for children with CNS tumours globally.

**Supplementary Information:**

The online version contains supplementary material available at 10.1007/s10143-026-04135-x.

## Introduction

Between 200,000 and 400,000 children are diagnosed with cancer each year globally [1]. Up to half of childhood cancer cases may be undiagnosed, particularly in low- and middle-income countries (LMICs) [2]. Despite a lower documented incidence of cancer in LMICs compared with high-income countries (HICs), total cancer-related mortality is significantly higher in LMICs [3]. It has been estimated that more than 80% of children with cancer in HICs are cured compared to less than 30% in LMICs, and recent studies have suggested that this disparity is worsening [4]. Cancer is a leading cause of non-accidental death among children worldwide [5].

Among childhood cancers, tumours of the brain and spinal cord – central nervous system (CNS) tumours – are the most common solid tumour type with an incidence of around 6 per 100,000 children [6]. CNS tumours are also the leading cause of death from cancer among children, and there is substantial morbidity among survivors [6, 7]. This morbidity is attributable either to the tumour’s location within the CNS or to the therapeutic interventions employed. The high frequency, mortality and morbidity underscore the global importance of optimising diagnosis, treatment, and rehabilitation of children with CNS tumours. Education and training efforts to reduce mortality and morbidity in childhood CNS tumours have focused on three areas: (1) minimising delays in recognising initial symptoms and signs suggestive of CNS tumours; (2) reducing diagnostic delay once a CNS tumour is suspected (collectively the latter two points are known as the symptom interval) [8–10], and (3) standardising treatment and rehabilitation protocols, which can vary widely both within and between countries [11]. Despite increasing awareness of these issues, there remains a paucity of robust data comparing the availability of diagnostic and treatment modalities for paediatric CNS tumours between and within healthcare facilities across income settings.

This study aims to collect facility-level data on the resources available for managing paediatric CNS tumours across countries of differing economic and development statuses to identify potential sources of disparities in care. Ultimately, these insights are intended to inform targeted interventions to enhance survival and reduce morbidity among children with CNS tumours globally.

## Methods

### Study design

This was a collaborative, international, questionnaire-based cross-sectional survey. A web-based survey hosted on the Qualtrics platform was disseminated using professional networks, neurosurgical mailing lists, and social media platforms to neurosurgeons and other physicians worldwide who were involved in the diagnosis and/or treatment of childhood CNS tumours. There are an estimated 2297 neurosurgeons who manage children, and therefore it is estimated that the total eligible population for this study is 3000 [12]. Participants were asked to provide informed consent for the use of their anonymised data in analyses and publications. Those who declined or were unable to provide consent could not proceed with the survey. Survey responses were collected between 16-Jan-2023 and 01-Feb-2024.

All aspects of this study were reviewed by the University of Oxford Ethics Board and were exempted from formal ethical approval requirements. All survey responses were anonymised, and no information was collected on individual patients or specific patient outcomes. Participants had the option to be named collaborators; any personal details provided were stored separately from survey responses.

### Survey

An initial pre-test questionnaire was designed based on existing literature related to the diagnosis and management of paediatric CNS tumours [13, 14]; no validated questionnaires were found. A paediatric neurosurgeon was defined as a neurosurgeon who had undertaken training and continued to perform surgeries mainly on children. A multidisciplinary team (MDT) meeting was defined as a regularly scheduled meeting for discussing children with CNS tumours attended by specialists from at least two different specialities. Availability in the survey referred to on-site capability as presented in supplemental content 1.

A paediatric neurosurgeon on each continent was invited to form part of a steering committee for this research. The pre-test questionnaire was piloted with this steering committee – who had not contributed to its original design – to gather direct feedback. Revisions were made to enhance clarity, ensure objectivity, and address potential sources of bias. A second round of feedback was conducted to produce the final questionnaire. The questionnaire was administered in English only, and comprised a combination of multiple-choice and free-text questions. Data were requested on participant demographics (including current role and years of training), healthcare facility characteristics, staffing (e.g., number of paediatric neurosurgeons), and details of diagnostic and treatment services (e.g. availability of imaging modalities). Respondents were asked to indicate the name or location of the centre in which they worked, although no identifying information about the centre (such as hospital name) was required if confidentiality was a concern. The full survey is available in supplemental content 1.

### Participants

**Fig. 1:**
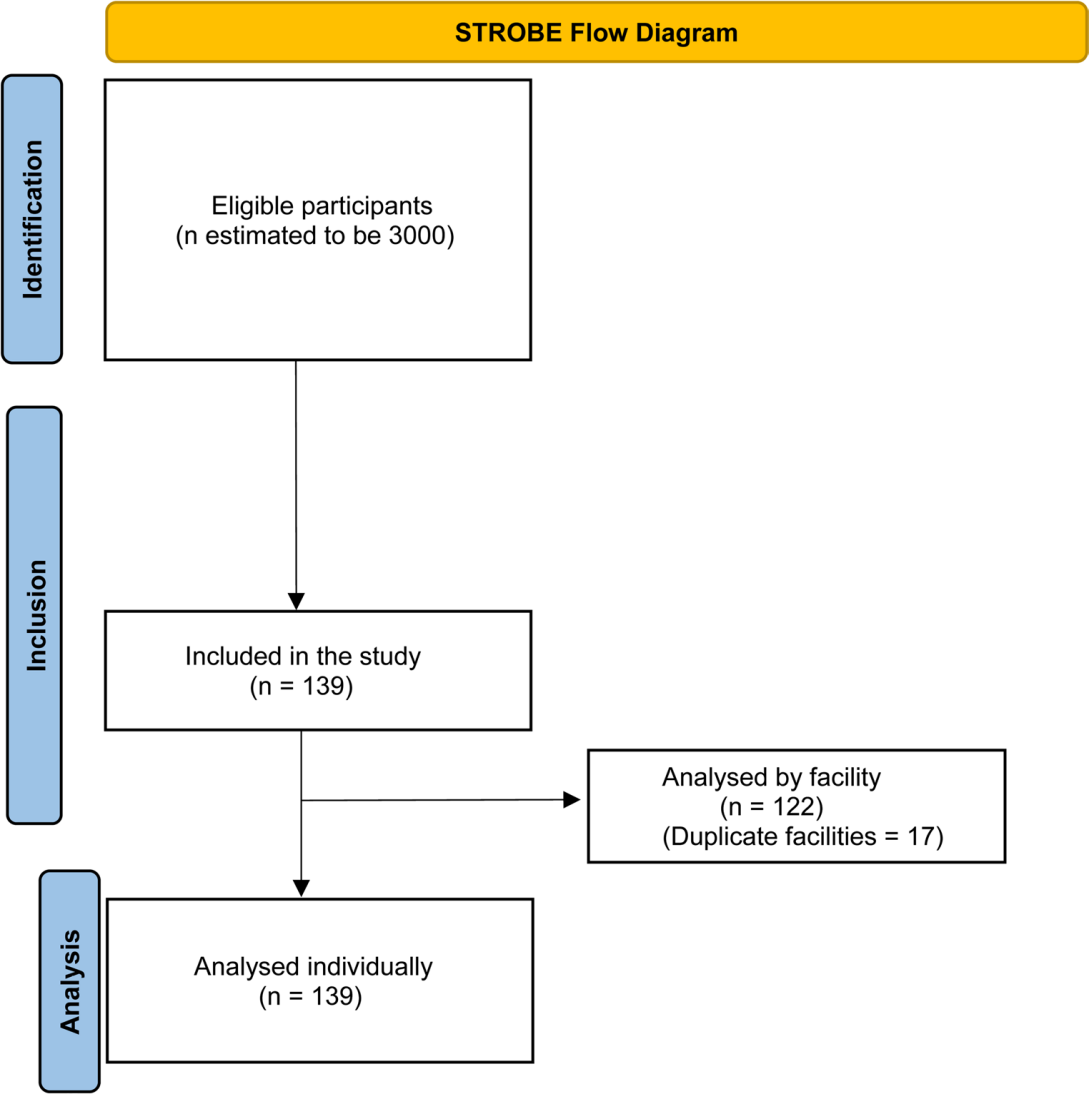
STROBE flow diagram of recruitment

Any physician involved in the diagnosis or management of CNS tumours worldwide was eligible to participate. This included specialist paediatric neurosurgeons, adult neurosurgeons, paediatric surgeons, and general surgeons who operated on neurosurgical conditions. Where surgeons were unable to answer facility-level data, other clinicians, such as neurologists or oncologists were approached. Where multiple participants originated from the same healthcare facility, facility-level data were cross-checked for consistency. When multiple respondents reported the same facility, item-level concordance was assessed; discordant items were set to missing for that facility, while concordant and more complete entries were retained. A STROBE flow diagram (Fig. 1) details recruitment, deduplication and analytic denominators.

### Statistical methods

Using each participant’s self-reported country of practice, responses were categorised by World Bank income level, United Nations Development Programme’s Human Development Index (HDI), Sustainable Development Index (SDI), and relevant geopolitical regions (e.g. Western Europe, North America, Asia-Pacific). When participants failed to answer a specific question or provided an invalid answer, that data point was excluded from the relevant analysis. The primary unit of analysis was the facility; respondent-level items are explicitly labelled. Proportions are presented with 95% confidence intervals (Wilson method; exact Clopper–Pearson intervals for sparse cells). Continuous variables are summarised using the median and interquartile range (IQR) unless otherwise specified. Differences in proportions were tested using χ² tests and Cochran–Armitage trend tests across ordered income tiers. Continuous non-normal variables were compared using Kruskal–Wallis tests. Statistical significance was set at *p* < 0.05. To address multiple comparisons across the primary availability endpoints, we applied Benjamini–Hochberg false discovery rate (FDR) control (q = 0.10). The values generated for binary outcomes were checked using: (i) generalised estimating equations (GEE) with a logit link and an exchangeable working correlation structure by country; and (ii) mixed-effects logistic regression with a random intercept for country; and for continuous outcomes, comparisons using linear mixed-effects models with a random intercept for country. These clustering-adjusted models yielded qualitatively unchanged inferences compared with the primary analyses. Analyses were conducted in R (v4.3) and Stata 18.

## Results

**Fig. 2:**
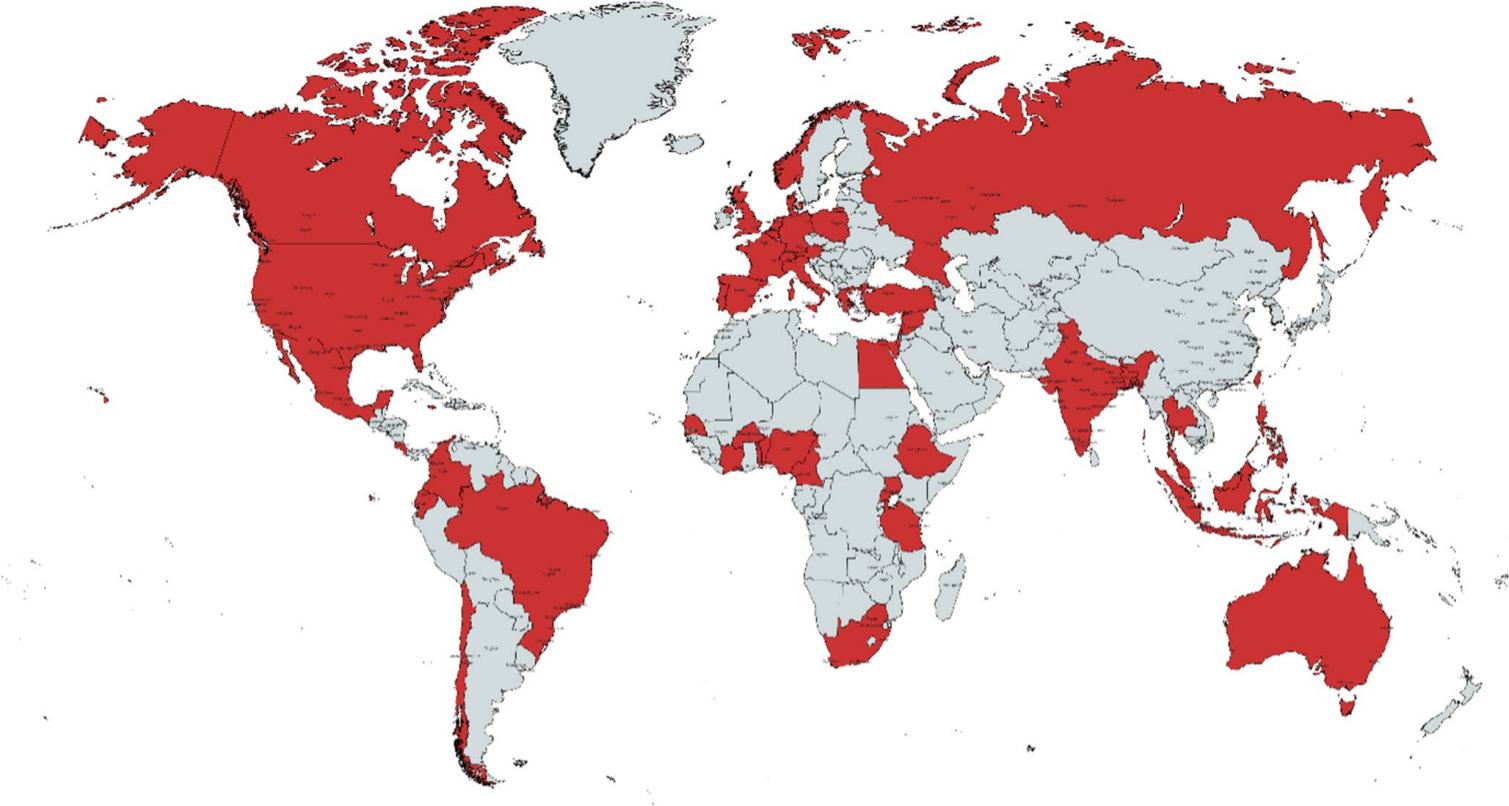
Countries of the survey respondents

A total of 139 clinicians responded to the survey, representing 122 different healthcare facilities across 110 cities or towns in 49 countries (Fig. 2, supplemental content 3). Most respondents were neurosurgical consultants (*n* = 120/138, 87.0%), followed by neurosurgical trainees or fellows (*n* = 11/138, 8.0%), paediatric surgeons (*n* = 5/138, 3.6%), a paediatric oncologist (*n* = 1/138, 0.7%), and a paediatric neurologist (*n* = 1/138, 0.7%).

Among neurosurgical consultants, 90 (75.0%) were paediatric neurosurgical consultants, of whom 25 (27.8%) operated exclusively on children. Of the adult neurosurgical consultants (*n* = 30), the 26 consultants (86.7%) who operated on paediatric patients all resided in LMICs. All paediatric surgeons (*n* = 5) also performed neurosurgical procedures on children. The median number of paediatric CNS tumours operated on by each clinician per year was 15 (IQR 10–30). There was no significant difference in the number of operations performed by the clinician’s country’s HDI, SDI, or income status.

**Fig. 3:**
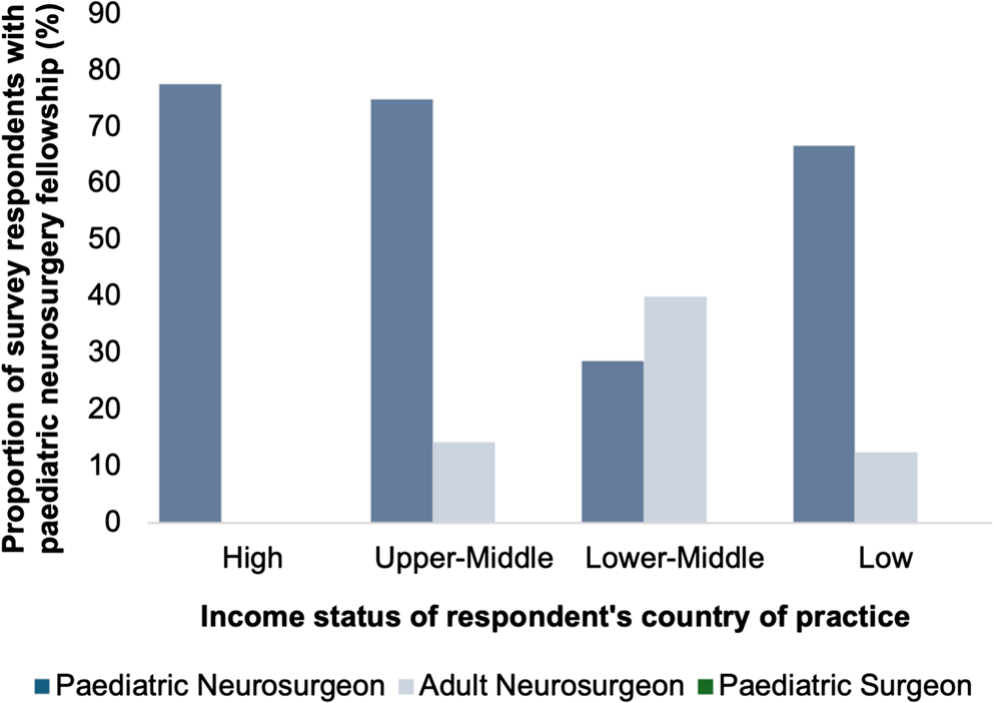
Proportion of surgeon survey respondents who undertook paediatric neurosurgery fellowships

Overall, neurosurgical consultants reported a median of seven years of training (IQR 6–9 years), with no significant differences between countries stratified by HDI, SDI, or income status. Most neurosurgical consultants (*n* = 70/118, 59.3% [95% CI: 50.3% − 67.8%]) had completed a formal paediatric neurosurgery fellowship, a proportion that rose to 73.0% (*n* = 65/89) [95% CI: 63.0% − 81.2%] among paediatric neurosurgery consultants. Fellowship attainment was lower among consultants practising in LMICs (Fig. 3; Table 1). Most neurosurgical consultants reported training in the same country in which they were currently practising. As shown in Table 2, the proportion of clinicians who trained domestically tended to decrease with declining HDI or SDI levels, although an exact pattern varied by index.

**Table 1:** Number and proportion of consultant neurosurgeons and paediatric surgeons who undertook paediatric neurosurgery fellowships

Designation of country		Paediatric Neurosurgeon (*n*, % of total)	Adult Neurosurgeon (*n*, % of total)	Paediatric Surgeon (*n*, % of total)
Human Development Index (HDI)	Very High	53 (77.9)	1 (33.3)	-
	High	8 (72.7)	1 (7.7)	-
	Medium	2 (33.3)	0 (0.0)	0 (0.0)
	Low	2 (50.0)	3 (30.0)	0 (0.0)
Sustainable Development Index (SDI)	High	52 (77.6)	0 (0.0)	-
	Medium	11 (61.1)	2 (11.8)	-
	Low	2 (50.0)	3 (30.0)	0 (0.0)
Income Status	High	52 (77.6)	0 (0.0)	-
	Upper-Middle	9 (75.0)	2 (14.3)	-
	Lower-Middle	2 (28.6)	2 (40.0)	0 (0.0)
	Low	2 (66.7)	1 (12.5)	0 (0.0)
Geopolitical Grouping	Western Europe	29 (69.1)	0 (0.0)	-
	North America	15 (100)	-	-
	Asia-Pacific	10 (52.6)	1 (7.7)	-
	Eastern Europe	4 (100)	-	-
	Latin America and the Caribbean	6 (100)	0 (0.0)	-
	Africa	1 (33.3)	4 (36.4)	0 (0.0)

**Table 2:** Location of neurosurgical training relative to current country of practice

Designation of country		Training and working in same country, *n* (%)	Training and working in different countries, *n* (%)
Human Development Index (HDI)	Very High	70 (92.1)	6 (7.9)
	High	20 (71.4)	8 (28.6)
	Medium	12 (100)	0 (0)
	Low	9 (50)	9 (50)
Sustainable Development Index (SDI)	High	67 (91.8)	6 (8.2)
	Medium	33 (80.5)	8 (19.5)
	Low	11 (55)	9 (45)

**Fig. 4:**
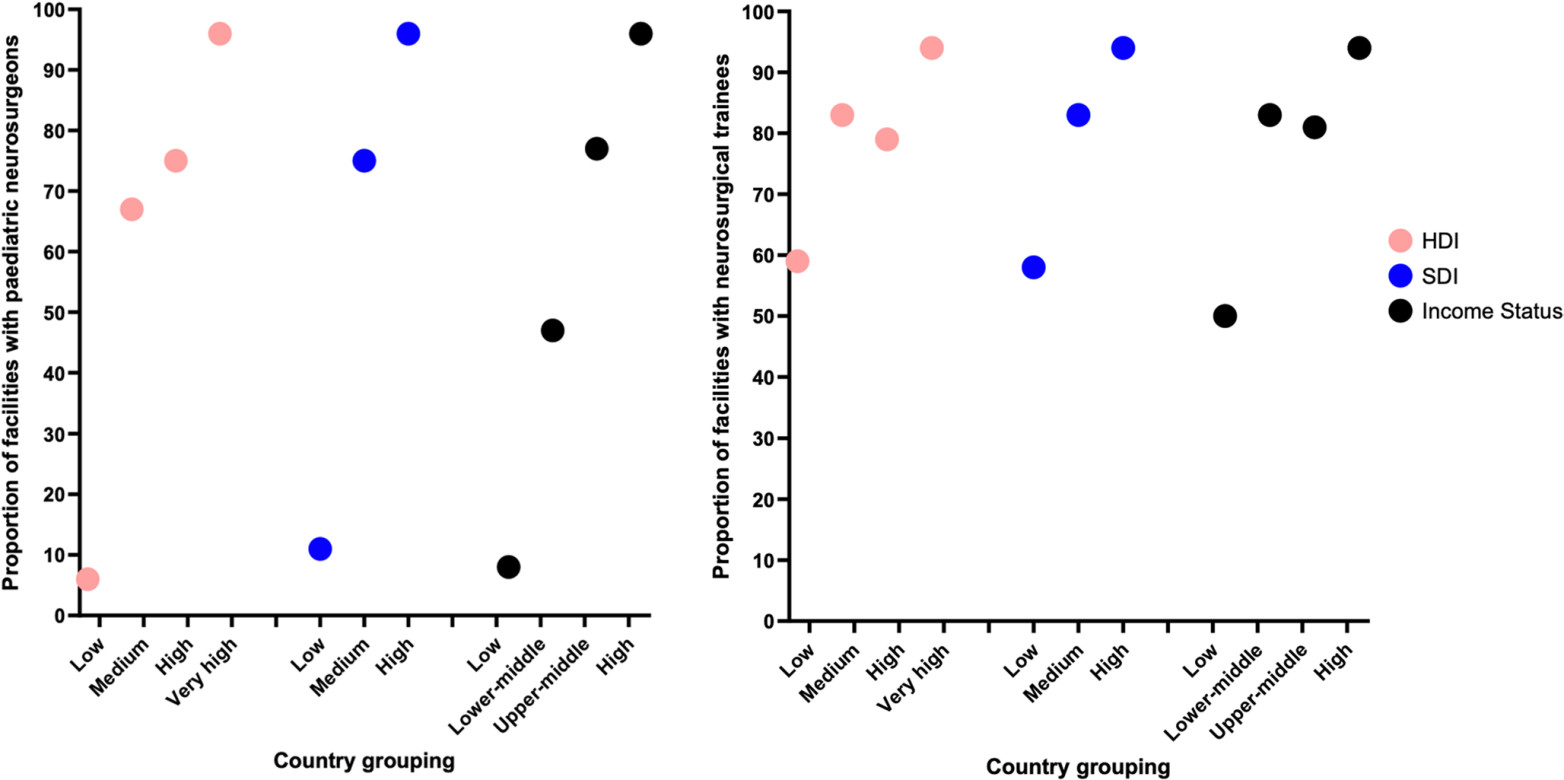
Proportion of facilities with paediatric neurosurgeons (left) and neurosurgical trainees (right) by World Bank income status, human development index (HDI) and sustainable development index (SDI)

Most respondents worked in tertiary hospitals (*n* = 113/122, 92.6% [95% CI: 86.6% − 96.1%]). Public hospitals were the most common workplace setting (*n* = 112/139, 80.6% [95% CI: 73.2% − 86.3%]); with 11 of these participants (9.8% [95% CI: 5.6% − 16.7%]) practising in both the public and private sector. There was no significant difference in workplace setting by HDI, SDI, or income status. A facility was more likely to have a paediatric neurosurgeon if it was located in a country with a higher HDI, SDI, or income status (*p* < 0.001; Fig. 4). Among centres with paediatric neurosurgeons, the median number of paediatric neurosurgeons was three (IQR 2–4), with no significant differences by HDI, SDI, or income status. By contrast, the presence of neurosurgical trainees was significantly associated with HDI, SDI, and income status (*p* = 0.001; Fig. 4).

Access to paediatric neurosurgery MDT meetings varied by income status. Almost all HIC facilities (*n* = 61/65, 93.9% [95% CI: 85.2% − 97.6%]) had an MDT, whereas only 40% (*n* = 4/10) [95% CI: 16.8% − 68.7%] of respondents from low-income countries reported having such meetings (*p* < 0.001). In HIC settings, MDTs were typically held weekly (*n* = 43/61, 70.5% [95% CI: 58.1% − 80.4%]), and 88.5% [95% CI: 78.2% − 94.3%] of facilities in these countries discussed all paediatric CNS tumours at each meeting. MDTs in LMICs were convened less frequently (most often monthly, *n* = 16/34, 47.1% [95% CI: 31.5% − 63.3%]), and not all paediatric CNS tumours were routinely discussed (*n* = 17/31, 54.8% [95% CI: 37.8% − 70.8%]).

**Fig. 5:**
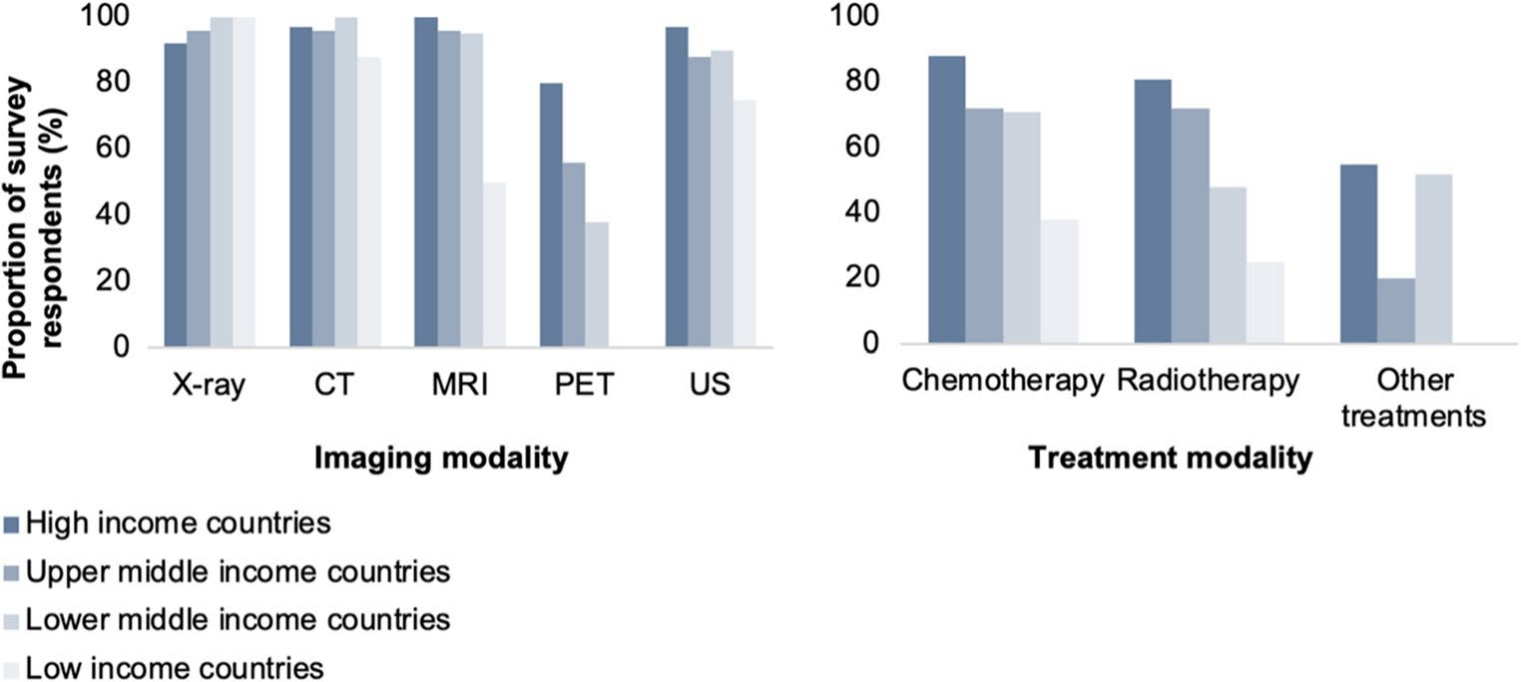
Availability of imaging (left) and treatment (right) modalities by income level

All participating centres had at least one radiologist employed; however, the availability of specialists varied. Of 115 healthcare facilities with data relating to this question, 84.5% (*n* = 96) [95% CI: 75.6% − 89.2%] had a neuroradiologist. Among 90 responses pertaining to different healthcare facilities, 67.8% (*n* = 61) had a paediatric neuroradiologist [95% CI: 57.6% − 76.5%]. Low-HDI countries were significantly less likely to have a neuroradiologist (*n* = 8/15, 53.3% [95% CI: 30.1% − 75.2%]) compared with those of medium to very high HDI (*n* = 88/100, 88.0% [95% CI: 80.2% − 93.0%]; *p* < 0.001). Basic imaging modalities (x-ray, CT, ultrasound) were widely available across income groups (Fig. 5). However, positron emission tomography (PET) was unavailable in any LIC (*p* < 0.001), and magnetic resonance imaging (MRI) was less common in LICs (*p* < 0.01).

Chemotherapy and radiotherapy services for paediatric CNS tumours showed similar disparities. Their availability was highest in HICs and progressively declined with lower country income level (*p* = 0.007 and *p* = 0.005, respectively; Fig. 5). The reported proportion of children followed up one year after treatment was highest in HICs (median 100%; IQR: 99% − 100%), followed by upper-middle-income countries (90%; IQR: 80% − 100%), lower-middle-income countries (67.5%; IQR: 50% − 75%), and low-income countries (34%; IQR: 10% − 60%). 73.4% and 34.5% of HIC and UMIC facilities respectively achieved 100% follow-up at one year.

## Discussion

This study provides evidence of a significant and pervasive discrepancy in the availability of resources for diagnosing and managing paediatric CNS tumours across countries of differing income and development statuses. Similar to other recent work in this field [13], our findings indicate that lower-income regions face particularly substantial shortfalls in trained paediatric neurosurgeons, advanced imaging technology, multidisciplinary management pathways, and essential oncological treatments. These deficits place children living in lower-income settings at a disadvantage and likely contribute to the poorer survival outcomes reported in LMICs. However, given the cross-sectional, self-reported design, findings indicate associations and should not be interpreted as causal.

Our data show that in many LMICs, adult neurosurgeons are treating children, which is broadly consistent with Baticulon et al. [14], who examined neurosurgery training in Asia and Australasia and found that neurosurgeons specialising in paediatrics frequently cared for adult patients. They explained this overlap with several reasons: small numbers of neurosurgeons serving large geographic areas, limited referral networks, and neurosurgeons with paediatric sub-specialty managing adult cases when no other surgeons are available. This scenario potentially limits the capacity of centres to provide optimal, specialised care for children with CNS tumours [15].

Beyond the variability in workforce structure, the survey revealed notable differences in training pathways. Over 90% of neurosurgeons from HICs reported sub-specialisation in paediatric neurosurgery compared to fewer than 20% of LIC respondents, and neurosurgeons in LMICs are more likely to have trained abroad. Reasons for these discrepancies may include insufficient domestic training schemes, financial barriers, and visa requirements for international fellowships. Although expanding fellowships abroad remains a plausible strategy for skill acquisition, there is a risk that practitioners may not return home to practise if opportunities, funding, or infrastructure remain inadequate. More sustainable approaches might therefore prioritise establishing in-country or regional training centres, facilitated by international mentorship and tele-education [16]. This can also include collaboration through international paediatric surgery societies and training initiatives, such as the Foundation for International Education in Neurological Surgery (FIENS). Locally embedded programmes can incorporate context-specific disease patterns, cultural factors, and healthcare pathways to help deliver effective services.

The provision of MDT care is another area where resource constraints and cultural factors intersect. Nearly all HIC centres in our survey had active MDTs for paediatric neurosurgery, yet only 40% of respondents from LICs reported similarly structured teams. The essential value of MDTs is well documented: they foster collaboration among neurosurgeons, oncologists, radiologists, pathologists, nurses, and allied professionals, enabling coordinated treatment strategies and timely follow-up. However, MDT functionality depends not only on having multiple specialists, but also on administrative support, robust referral systems, adequate operating theatre resources, and a reliable supply of consumables [17]. In LMICs, even partial or informal MDT structures could improve care, for example by holding weekly or monthly teleconferences between district hospitals and tertiary centres to discuss complex cases [18].

Diagnostic imaging emerged as another critical bottleneck. In HICs, MRI and PET are routine elements of neurosurgical management, whereas their availability in LMICs is sporadic. These advanced imaging modalities are crucial for evaluating tumour location, delineating surgical margins, and monitoring recurrence. The inability to obtain detailed imaging may contribute to delayed or incomplete diagnoses, and to less precise surgical resections that can lead to poorer outcomes. The equipment may be available in some settings but not accessible due to location or costs. Possible solutions may include optimising existing CT protocols, training local radiographers, and establishing reliable referral pathways to centres equipped with MRI [19]. Although mobile MRI services or cross-border referrals may help, these solutions require coordinated investments in maintenance, staffing, and infrastructure.

Non-surgical therapeutic options – reported by a predominantly neurosurgical sample (including only one paediatric oncologist) – showed similarly stark disparities. Although there may have been misclassification of non-surgical services given the sample population, patterns observed for chemotherapy and radiotherapy availability are concordant with prior oncology-led assessments [13, 20]. These show that treatment abandonment, delays, or incomplete regimens disproportionately affect children in LMICs. Governments, non-governmental organisations, and philanthropic institutions could help by subsidising radiotherapy facilities or working with private-sector providers to lower treatment costs. Strengthening chemotherapy supply chains and standardising protocols around common, cost-effective agents might improve access and reduce unpredictable drug availability. Cultural and socio-economic considerations are integral to these interventions [21], particularly in contexts where cancer remains stigmatised or poorly understood. Families may rely on traditional healers, and financial constraints often lead to incomplete treatment courses [9]. To mitigate high costs, creative financing solutions such as microinsurance, community-based health funds, or travel subsidies for families are essential. These steps are more feasible when local health ministries and international partners set childhood cancer as a priority [12–14, 22].

Expanding reliable epidemiological data from LMICs is similarly crucial. Accurate cancer registries are instrumental in determining disease burden, allocating resources, and measuring the efficacy of interventions [23]. Collaborative research networks could also examine the viability of telemedicine-based MDT meetings, teleradiology, or region-specific approaches to post-operative care. Such studies would help identify best practices that are feasible in low-resource environments, rather than simply importing high-income models of care.

Although this study’s findings are robust in illustrating the scope of global disparities, certain limitations must be acknowledged. As a cross-sectional survey, it provides a single time-point view and cannot capture dynamic changes in healthcare infrastructure, and is prone to recall bias. Given multiple comparisons were conducted, there is a risk of a Type 1 error in the multiple p values that have been generated; controlling the false discovery rate (q = 0.10) yielded inferences consistent with unadjusted analyses. Voluntary participation, combined with an English-language questionnaire, introduces the possibility of selection and non-response bias, potentially underrepresenting smaller centres or clinicians less connected to international research networks. However, our survey reached a broader range of clinicians than existing studies, including those not necessarily affiliated with paediatric neurosurgical societies. Moreover, we were unable to analyse data at a national or regional level, due to confidentiality requirements and variations in response rates. Sensitivity analyses accounting for clustering by country (GEE and mixed-effects models) produced qualitatively unchanged conclusions for primary endpoints. Nevertheless, by employing World Bank income classifications and cross-referencing with the HDI and SDI, we drew meaningful comparisons across socio-economic strata. One distinguishing feature of our study is that it separately categorises LMICs rather than combining them. This method allows a more refined analysis of how factors such as national wealth and healthcare spending influence service availability. The survey did not elicit information on healthcare financing, information systems, access to devices, and governance, which influence the provision of high-quality surgical care. Similarly, non-surgical service availability was clinician-reported by predominantly neurosurgical respondents, and this may imperfectly capture oncology service realities. Finally, without patient-specific outcomes or other clinical end-points, we cannot directly correlate resource availability with survival or quality of life, although existing literature strongly suggests that consistent access to surgery, imaging, and oncological therapies is essential to reducing mortality.

Despite these constraints, this study augments and corroborates previous surveys on global paediatric neurosurgical capacity, emphasising how inequalities in training, infrastructure, and service coordination can have profound consequences for children with CNS tumours. Policymakers could prioritise the establishment of regional centres of excellence, incentivise local fellowship programmes, and create referral networks that ensure no child is excluded from timely and effective treatment. The WHO Global Initiative for Childhood Cancer aims to increase survival rates in LMICs from 20% to 60% by 2030 [24], but achieving this ambitious goal will require a concerted effort that balances high-level advocacy with practical, on-the-ground solutions tailored to each country’s economic, cultural, and structural realities.

## Supplementary Information

Below is the link to the electronic supplementary material.

Supplementary Material 1 Supplemental content 1 – Survey: Availability of services to manage paediatric CNS tumours globally

Supplementary Material 2 Supplemental content 2 – Sensitivity analyses

Supplementary Material 3 Supplemental content 3 – Location of survey respondents

Supplementary Material 4 Supplemental content 4 – List of collaborative authors

## Data Availability

The datasets used and/or analysed during the current study are available from the corresponding author on reasonable request.

## Abbreviations

LMIC: Low-or Middle-Income Country
HIC: High-Income Country
CNS: Central Nervous System
MDT: Multidisciplinary Team
HDI: Human Development Index
SDI: Sustainable Development Index
ANOVA: Analysis Of Variance
IQR: Interquartile Range
LIC: Low Income Country
PET: Positron Emission Tomography
MRI: Magnetic Resonance Imaging
WHO: World Health Organisation
