# Correction to: Neurosurgical management of paediatric central nervous system tumours in low, middle and high-income countries: a multi-centre, international, cross-sectional study

**DOI:** 10.1007/s10143-026-04204-1

**Published:** 2026-03-10

**Authors:** Solange Bramer, Soham Bandyopadhyay, Ruth Mitchell, Andreas K. Demetriades, Ronnie E. Baticulon, Jogi Pattisapu, Andres Rubiano, Nqobile Thango, Kokila Lakhoo, Global Children’s NCDs Collaborative

**Affiliations:** 1 Nuffield Department of Surgical Sciences, Oxford University Global Surgery Group, University of Oxford, Oxford, UK; 2 Clinical Neurosciences, Clinical & Experimental Sciences, Faculty of Medicine, University of Southampton, Southampton, Hampshire UK; 3 Wessex Neurological Centre, University Hospital Southampton NHS Foundation Trust, Southampton, UK; 4 Department of NeurosurgeryNew South Wales Health, Sydney Children’s Hospital, Randwick, NSW Australia; 5 Department of Neurosurgery, Royal Infirmary Edinburgh, Edinburgh, Scotland UK; 6 Leiden University Medical Centre, Leiden, The Netherlands; 7 Division of Neurosurgery, Department of Neurosciences, Philippine General Hospital, University of the Philippines Manila, Manila, Philippines; 8 College of Medicine, University of Central Florida, Orlando, Fl USA; 9 Neuroscience Institute, Universidad El Bosque, Bogota, Colombia; 10 Division of Neurosurgery, Department of Surgery, University of Cape Town, Cape Town, South Africa; 11 Neuroscience Institute, University of Cape Town, Cape Town, South Africa

**Correction to: Neurosurgical Review**

10.1007/s10143-026-04135-x

The authors regret that that the name of Andreas K. Demetriades was incorrectly captured as Andreas Demetriades in the original published article. It is now corrected in this erratum article.

The original article online has been corrected.

