# Supplementary material for: Neurosurgical management of paediatric central nervous system tumours in low, middle and high-income countries: a multi-centre, international, cross-sectional study
Source: Neurosurg Rev. 2026 Jan 31;49(1):185. doi: 10.1007/s10143-026-04135-x (PMC12860862; doi:10.1007/s10143-026-04135-x)
Supplement: Supplementary file 2 — Supplementary Material 2 Supplemental content 2 – Sensitivity analyses [file 10143_2026_4135_MOESM2_ESM.docx]

**APPENDIX 1**

**Survey: Availability of services to manage paediatric brain tumours globally**

**Introduction**

Brain tumours are the second most common paediatric tumour and are the leading cause of cancer death in children. Of the children that survive brain tumours, there is considerable morbidity with many children being left with life-altering disability. This may be a direct consequence of the nature of childhood brain tumours or may be attributed to their management. 

We are conducting a study to help understand how patients with paediatric brain tumours are diagnosed and treated at different centres across the world.

Despite a lower documented incidence of paediatric cancers in low- and middle-income countries (LMICs), total cancer-related mortality is significantly higher in LMICs compared to high-income countries (HICs). It is estimated that more than 80% of children with cancer in HICs are cured, compared to less than 30% in LMICs. 

There is a paucity of data comparing paediatric brain tumour presentation, diagnosis and management between high, middle and low-income countries. The available literature suggests wide discrepancies in the infrastructure, availability of diagnostic techniques and treatment modalities available to children with brain tumours. The aims of this study are to explore the disparities in the management of paediatric brain tumours both between geographically different centres and centres located in HICs and LMICs. 

We are disseminating this survey to surgeons worldwide. The questions focus on the diagnostic and therapeutic methods available for brain tumour management. No patient identifiable will be collected and all survey responses are anonymous. Once data collection is complete, the survey responses will be freely available. All survey respondents will become official named collaborators on any research work that stems from this work. There is one question at the end of the survey asking for name and institution of survey respondent which is solely used to name the authors when publishing work stemming from this study, and will not be used when extracting and analysing the survey responses. 

If you have any questions about the content of this survey, about the study in general, or would like to receive updates about the study, then please contact either Dr Solange Bramer (solange.bramer@mkuh.nhs.uk) or Dr Soham Bandyopadhyay (soham.bandyopadhyay@st-hildas.ox.ac.uk).

**Consent**

This survey will take approximately 5 minutes to complete. The majority of questions are multiple choice. Questions asking for numbers (ie number of brain tumours per year at your centre) do not require official values but rather an estimate. No personal identifiable information is being collected for research purposes. The last question asks for your name and institution so you can be a named collaborator on any research outputs from this data and this information will not be analysed together with the rest of the data. This question is optional and you do not need to answer it if you do not wish to be a named collaborator. All other data collected through this survey will be collated and analysed for research purposes.

*Please tick here if you consent to take part in this study.*

**Characteristics of centre**

1. Which country do you work in? *Drop-down list of every country*
2. What is the name of the city/town your hospital is located in? ________
3. Do you work at public or private hospitals?
   1. Public hospital
   2. Private hospital
   3. Both
4. Which kind of hospital do you work at? Here, a secondary hospital is a hospital that provides general medical and surgical services. A tertiary hospital is a hospital that provides highly specialist treatment. Please tick all that apply.
   1. Secondary hospital
   2. Tertiary hospital
   3. Other: ________
5. Is paediatric neurosurgery performed at your centre?
   1. Yes
   2. No
   3. I don’t know
6. *(If ‘yes’ to question 5)* Who performs paediatric neurosurgery at your centre? Please tick all that apply.
   1. Adult general surgeons
   2. Adult neurosurgeons
   3. Paediatric surgeons
   4. Paediatric neurosurgeons
   5. Other: _______
   6. Paediatric neurosurgery is not performed at my centre
7. Are there paediatric neurosurgeons at your centre?
   1. Yes
   2. No
   3. I don’t know
8. *(If ‘yes’ to question 7)* Approximately how many paediatric neurosurgeons work at this centre? ____________
9. *(If ‘yes’ to question 7)*  Are there any fellowship-trained paediatric neurosurgeons at this centre? Here, a fellowship refers to a post in paediatric neurosurgery of at least 6 months undertaken after standard surgical training.
   1. Yes
   2. No
   3. I don’t know
10. *(If ‘yes’ to question 8)* Approximately how many fellowship-trained paediatric neurosurgeons work at this centre? ____________
11. Are there neurosurgery residents/registrars/fellows training at your centre?
    1. Yes
    2. No
    3. I don’t know

**Characteristics of neurosurgeon**

1. Are you a neurosurgery consultant/attending or trainee?
   1. Neurosurgery consultant/attending
   2. Neurosurgery trainee
   3. Other: _______________
2. (*If ‘yes’ to question 11)* Approximately how long was your neurosurgical training? (in years). Here, neurosurgical training is defined as the years between finishing medical school and becoming a consultant/attending. Please exclude any years of non-clinical work.
3. Do you (sub)specialise in paediatric neurosurgery?
   1. Yes
   2. No
4. Do you perform neurosurgery on adults and children?
   1. Yes, I operate on both adults and children
   2. No, only on adults
   3. No, only on children
5. Where did you attend the majority of your neurosurgery training? *Drop-down list of all countries*
6. Did you undertake a fellowship in paediatric neurosurgery? Here, a fellowship refers to a post in paediatric neurosurgery of at least 6 months undertaken after standard surgical training.
   1. Yes
   2. No
7. *(If ‘yes’ is answered to question 17)* Where did you undertake your fellowship in paediatric neurosurgery? Please state the city and country. _________
8. Do you operate independently (without supervision) on paediatric brain tumours?
   1. Yes
   2. No
9. *(If ‘yes’ to question 19)* How long have you been operating independently (without supervision) on brain tumours (in years)? __________
10. Approximately how many paediatric brain tumours do you operate on every year? ________

**Diagnosis of paediatric brain tumours**

1. How are patients with paediatric brain tumours referred to your care/centre? Please tick all that apply.
   1. General practice (GP)
   2. Paediatricians
   3. Other specialties e.g. ophthalmologists, neurologists
   4. Self-referral
   5. Other hospitals
   6. Other neurosurgery centres
   7. Other: ___________
2. What imaging techniques are available at your centre? (Tick all that apply)
   1. X-ray
   2. Computerised tomography (CT)
   3. Magnetic resonance imaging (MRI)
   4. Positron emission tomography (PET)
   5. Ultrasound
   6. Other: ______________
3. Are there radiologists at your centre?
   1. Yes
   2. No
   3. I don’t know
4. *(If ‘yes’ answered to question 24)* Are there neuroradiologists at your centre?
   1. Yes
   2. No
   3. I don’t know
5. *(If ‘yes’ answered to question 25)* Are there paediatric neuroradiologists at your centre?
   1. Yes
   2. No
   3. I don’t know
6. Would you operate on a paediatric brain tumour without a radiologist’s opinion or official radiology report?
   1. Yes, in both emergency and elective cases
   2. Yes, but only in an emergency
   3. No
7. *(If ‘yes, in both emergency and elective cases’ or ‘yes, but only in an emergency’ answered to question 27*) Have you received formal training in interpreting scans for paediatric brain tumours?
   1. Yes
   2. No
8. Do you have the option of discussing challenging paediatric brain tumour cases with colleagues at other centres? Please tick all that apply.
   1. Yes, with colleagues in the same country
   2. Yes, with colleagues in other countries
   3. No
9. Are there multidisciplinary team (MDT) meetings for paediatric neurosurgery at your centre? Here, MDT is defined as a meeting attended by doctors belonging to at least three different specialties.
   1. Yes
   2. No
10. *(If ‘yes’ answered to question 30)* How frequently do multidisciplinary team (MDT) meetings take place?
    1. Every week
    2. Every month
    3. Quarterly (every three months)
    4. Every six months
    5. Every year
    6. It varies
11. Which healthcare professionals are invited to attend these MDT meetings? Please tick all that apply.

- General surgeon
- Paediatric surgeon
- Neurosurgeon
- Neurologist
- General oncologist
- Paediatric oncologist
- Specialist cancer nurse
- Radiologist
- Radio-oncologist
- Pathologist
- Paediatrician
- Other allied healthcare professionals, i.e. physiotherapists
- Other: _________

1. Does every paediatric brain tumour that present at your centre get discussed at a multidisciplinary team (MDT) meeting at your hospital?
   1. Yes
   2. No
   3. I don’t know
   4. Prefer not to say
2. *(If ‘no’ is answered to question 33)* Are treatment plans for patients ever decided by one doctor?
   1. Yes
   2. No
   3. I don’t know
   4. Prefer not to say
3. *(If ‘no’ is answered to question 34)* How are treatment plans decided for paediatric brain tumours at your centre? _________

**Types of paediatric brain tumours**

1. Please pick the three paediatric brain tumours you care for most frequently.
   1. Low-grade glioma
   2. Craniopharyngioma
   3. Germ cell tumour
   4. Malignant glioma
   5. Medulloblastoma
   6. Ependymoma
   7. Choroid plexus tumour

**Management of paediatric brain tumours**

1. Do children with brain tumours undergo chemotherapy at your centre?
   1. Yes
   2. No
   3. I don’t know
2. Do children with brain tumours undergo radiotherapy at your centre?
   1. Yes
   2. No
   3. I don’t know
3. Are there other treatments available for paediatric brain tumours at your centre (treatments other than surgery, chemotherapy and radiotherapy)?
   1. Yes
   2. No
   3. I don’t know
4. *(If ‘yes’ answered to question 39*) Please state which other treatments are available for children with brain tumours at your centre. ______________
5. Do you ever refer children with brain tumours to other centres?
   1. Yes
   2. No
   3. Prefer not to say
6. *(If ‘yes’ answered to question 41)* Why do you refer children with brain tumours to other centres? (Please tick all that apply)
   1. Expertise at other centres
   2. Second opinion
   3. Patient/family choice
   4. Limited diagnostic capacity at own centre
   5. Limited treatment capacity at own centre
   6. Limited treatment options available at own centre
   7. Other: __________
7. What percentage of patients are followed-up for at least a year after surgery? Please give a number between 0 and 100. _________

**Becoming a named collaborator**

Please click on the following link address if you would like to become a named collaborator on any publications/presentations that stem from this study. The survey answers are not linked and cannot be traced back to your email address.
