# Supplementary material for: Neurosurgical management of paediatric central nervous system tumours in low, middle and high-income countries: a multi-centre, international, cross-sectional study
Source: Neurosurg Rev. 2026 Jan 31;49(1):185. doi: 10.1007/s10143-026-04135-x (PMC12860862; doi:10.1007/s10143-026-04135-x)
Supplement: Supplementary file 3 — Supplementary Material 3 Supplemental content 3 – Location of survey respondents [file 10143_2026_4135_MOESM3_ESM.docx]

**Supplemental content 2: Location of survey respondents**

| Country | Town/City | Number of Responses | Human Development Index (HDI) | Sustainable Development Index (SDI) | Income Status | Geopolitical Grouping | Developmental Status |
| --- | --- | --- | --- | --- | --- | --- | --- |
| Australia | Brisbane | 1 | Very high | High | High | Asia-Pacific | Developed |
| Australia | Sydney | 1 | Very high | High | High | Asia-Pacific | Developed |
| Australia | Melbourne | 2 | Very high | High | High | Asia-Pacific | Developed |
| Austria | Vienna | 1 | Very high | High | High | Western Europe | Developed |
| Austria | Linz | 2 | Very high | High | High | Western Europe | Developed |
| Bangladesh | Dhaka | 1 | Medium | Low | Lower-middle | Asia-Pacific | Developing |
| Belgium | Brussels | 1 | Very high | High | High | Western Europe | Developed |
| Belgium | Leuven | 3 | Very high | High | High | Western Europe | Developed |
| Benin | Cotonou | 1 | Low | Low | Low | Africa | Developing |
| Benin | Parakou | 1 | Low | Low | Low | Africa | Developing |
| Brazil | Recife | 1 | High | Medium | Upper-middle | Latin America and the Caribbean | Developing |
| Brazil | Sao Paulo | 1 | High | Medium | Upper-middle | Latin America and the Caribbean | Developing |
| Burkina Faso | Bobo-Dioulasso | 1 | Low | Low | Low | Africa | Developing |
| Burkina Faso | Tenkodogo | 1 | Low | Low | Low | Africa | Developing |
| Burkina Faso | Ouagadougou | 2 | Low | Low | Low | Africa | Developing |
| Cameroon | Garoua | 1 | Low | Low | Lower-middle | Africa | Developing |
| Canada | Calgary | 1 | Very high | High | High | North America | Developed |
| Canada | London, Ontario | 1 | Very high | High | High | North America | Developed |
| Canada | Montreal | 1 | Very high | High | High | North America | Developed |
| Canada | Toronto | 1 | Very high | High | High | North America | Developed |
| Canada | Halifax | 2 | Very high | High | High | North America | Developed |
| Canada | Ottawa | 2 | Very high | High | High | North America | Developed |
| Chile | Concepcion | 1 | Very high | High | High | Latin America and the Caribbean | Developed |
| Colombia | Bogota | 1 | High | Medium | Upper-middle | Latin America and the Caribbean | Developing |
| Colombia | Bolívar | 1 | High | Medium | Upper-middle | Latin America and the Caribbean | Developing |
| Colombia | Cali | 1 | High | Medium | Upper-middle | Latin America and the Caribbean | Developing |
| Costa Rica | San Jose | 1 | High | Medium | Upper-middle | Latin America and the Caribbean | Developing |
| Denmark | Aarhus | 1 | Very high | High | High | Western Europe | Developed |
| Denmark | Copenhagen | 2 | Very high | High | High | Western Europe | Developed |
| Ecuador | Cuenca | 1 | High | Medium | Upper-middle | Latin America and the Caribbean | Developing |
| Ecuador | Quito | 1 | High | Medium | Upper-middle | Latin America and the Caribbean | Developing |
| Egypt | Cairo | 1 | Medium | Low | Lower-middle | Africa | Developing |
| Ethiopia | Addis Ababa | 2 | Low | Low | Low | Africa | Developing |
| France | Lyon | 1 | Very high | High | High | Western Europe | Developed |
| Germany | Frankfurt am Main | 1 | Very high | High | High | Western Europe | Developed |
| Germany | Freiburg | 1 | Very high | High | High | Western Europe | Developed |
| Germany | Hamburg | 1 | Very high | High | High | Western Europe | Developed |
| Germany | Rostock | 1 | Very high | High | High | Western Europe | Developed |
| Germany | Sankt Augustin | 1 | Very high | High | High | Western Europe | Developed |
| Germany | Mainz | 2 | Very high | High | High | Western Europe | Developed |
| Greece | Thessaloniki | 2 | Very high | High | High | Western Europe | Developed |
| Guinea | Conakry | 1 | Low | Low | Low | Africa | Developing |
| India | Bengaluru | 1 | Medium | Medium | Lower-middle | Asia-Pacific | Developing |
| India | Chandigarh | 1 | Medium | Medium | Lower-middle | Asia-Pacific | Developing |
| India | Delhi | 1 | Medium | Medium | Lower-middle | Asia-Pacific | Developing |
| India | Hyderabad | 1 | Medium | Medium | Lower-middle | Asia-Pacific | Developing |
| India | Mumbai | 1 | Medium | Medium | Lower-middle | Asia-Pacific | Developing |
| India | New Delhi | 1 | Medium | Medium | Lower-middle | Asia-Pacific | Developing |
| India | Vellore | 1 | Medium | Medium | Lower-middle | Asia-Pacific | Developing |
| India | Visakhapatnam | 1 | Medium | Medium | Lower-middle | Asia-Pacific | Developing |
| Indonesia | Bandung | 1 | High | Medium | Upper-middle | Asia-Pacific | Developing |
| Indonesia | Jakarta | 1 | High | Medium | Upper-middle | Asia-Pacific | Developing |
| Israel | Tel Aviv | 2 | Very high | High | High | Western Europe | Developed |
| Italy | Firenze | 1 | Very high | High | High | Western Europe | Developed |
| Italy | Messina | 1 | Very high | High | High | Western Europe | Developed |
| Italy | Monza | 1 | Very high | High | High | Western Europe | Developed |
| Italy | Padova | 1 | Very high | High | High | Western Europe | Developed |
| Italy | Rome | 1 | Very high | High | High | Western Europe | Developed |
| Italy | Milano | 2 | Very high | High | High | Western Europe | Developed |
| Ivory Coast | Abidjan | 1 | Low | Low | Lower-middle | Africa | Developing |
| Jamaica | Kingston | 2 | High | Medium | Upper-middle | Latin America and the Caribbean | Developing |
| Lebanon | Beirut | 1 | High | Medium | Upper-middle | Asia-Pacific | Developing |
| Malaysia | Kuala Lumpur | 3 | High | Medium | Upper-middle | Asia-Pacific | Developing |
| Mexico | Monterrey | 1 | High | Medium | Upper-middle | Latin America and the Caribbean | Developing |
| Netherlands | Nijmegen | 1 | Very high | High | High | Western Europe | Developed |
| Nigeria | Ibadan | 1 | Low | Low | Lower-middle | Africa | Developing |
| Nigeria | Umuahia | 2 | Low | Low | Lower-middle | Africa | Developing |
| Norway | Bergen | 1 | Very high | High | High | Western Europe | Developed |
| Norway | Oslo | 1 | Very high | High | High | Western Europe | Developed |
| Philippines | City of San Fernando | 1 | Medium | Medium | Lower-middle | Asia-Pacific | Developing |
| Philippines | Manila | 1 | Medium | Medium | Lower-middle | Asia-Pacific | Developing |
| Poland | Krakow | 1 | Very high | High | High | Eastern Europe | Developed |
| Poland | Katowice | 2 | Very high | High | High | Eastern Europe | Developed |
| Portugal | Lisboa | 1 | Very high | High | High | Western Europe | Developed |
| Russian Federation | Moscow | 2 | Very high | Medium | Upper-middle | Eastern Europe | Developed |
| Senegal | Dakar | 1 | Low | Low | Lower-middle | Africa | Developing |
| South Africa | Lusaka | 2 | High | Medium | Upper-middle | Africa | Developing |
| Spain | Barcelona | 1 | Very high | High | High | Western Europe | Developed |
| Spain | Malaga | 1 | Very high | High | High | Western Europe | Developed |
| Spain | Palma de Mallorca | 1 | Very high | High | High | Western Europe | Developed |
| Spain | Santander | 1 | Very high | High | High | Western Europe | Developed |
| Switzerland | Basel | 1 | Very high | High | High | Western Europe | Developed |
| Switzerland | Bern | 1 | Very high | High | High | Western Europe | Developed |
| Switzerland | Lausanne | 1 | Very high | High | High | Western Europe | Developed |
| Syrian Arab Republic | Damascus | 1 | Low | Low | Low | Asia-Pacific | Developing |
| Taiwan | Taichung | 1 | Very high | High | High | Asia-Pacific | Developed |
| Taiwan | Yunlin | 1 | Very high | High | High | Asia-Pacific | Developed |
| Taiwan | Taipei | 4 | Very high | High | High | Asia-Pacific | Developed |
| Thailand | Hat Yai | 1 | High | Medium | Upper-middle | Asia-Pacific | Developing |
| Thailand | Bangkok | 3 | High | Medium | Upper-middle | Asia-Pacific | Developing |
| Thailand | Pathum Thani | 5 | High | Medium | Upper-middle | Asia-Pacific | Developing |
| Togo | Lomé | 1 | Low | Low | Low | Africa | Developing |
| Turkey | Denizli | 1 | Very high | Medium | Upper-middle | Asia-Pacific | Developing |
| Uganda | Mbarara | 1 | Low | Low | Low | Africa | Developing |
| United Kingdom of Great Britain and Northern Ireland | Birmingham, UK | 1 | Very high | High | High | Western Europe | Developed |
| United Kingdom of Great Britain and Northern Ireland | Leeds | 1 | Very high | High | High | Western Europe | Developed |
| United Kingdom of Great Britain and Northern Ireland | Liverpool | 1 | Very high | High | High | Western Europe | Developed |
| United Kingdom of Great Britain and Northern Ireland | London | 1 | Very high | High | High | Western Europe | Developed |
| United Kingdom of Great Britain and Northern Ireland | Oxford | 1 | Very high | High | High | Western Europe | Developed |
| United Kingdom of Great Britain and Northern Ireland | Southampton | 1 | Very high | High | High | Western Europe | Developed |
| United Republic of Tanzania | Dar es salaam | 1 | Low | Low | Low | Africa | Developing |
| United States of America | Aurora | 1 | Very high | High | High | North America | Developed |
| United States of America | Birmingham, USA | 1 | Very high | High | High | North America | Developed |
| United States of America | Indianapolis | 1 | Very high | High | High | North America | Developed |
| United States of America | Memphis | 1 | Very high | High | High | North America | Developed |
| United States of America | Milwaukee | 1 | Very high | High | High | North America | Developed |
| United States of America | Newark | 1 | Very high | High | High | North America | Developed |
| United States of America | Orlando | 1 | Very high | High | High | North America | Developed |
| United States of America | Rochester | 1 | Very high | High | High | North America | Developed |
| United States of America | Syracuse | 1 | Very high | High | High | North America | Developed |
