# Supplementary material for: Neurosurgical management of paediatric central nervous system tumours in low, middle and high-income countries: a multi-centre, international, cross-sectional study
Source: Neurosurg Rev. 2026 Jan 31;49(1):185. doi: 10.1007/s10143-026-04135-x (PMC12860862; doi:10.1007/s10143-026-04135-x)
Supplement: Supplementary file 4 — Supplementary Material 4 Supplemental content 4 – List of collaborative authors [file 10143_2026_4135_MOESM4_ESM.docx]

**Collaborative authors** (medical students/doctors who helped collect survey responses and survey respondents)

| **First name** | **Last name** | **Affiliation** |
| --- | --- | --- |
| Julia R | Costa | Faculdade Ciências Médicas de Minas Gerais, Belo Horizonte, Brazil |
| Monique BM | Gomes | Federal University of Piauí, Piaui, Brazil |
| Zekai | Qiang | School of Medicine and Population Health, University of Sheffield Medical School, Sheffield, UK |
| Miho | Shinozuka | Fukushima Medical University, Fukushima, Japan |
| Yvan | Zolo | Global Surgery Division, University of Cape Town, Cape Town, South Africa |
| Dr Nunthasiri | Wittayanakorn | Queen Sirikit National Institute of Child Health, Bangkok, Thailand |
| Dr Mukesch Johannes | Shah | Klinik für Neurochirurgie, Universitatsklinikum Freiburg, Germany |
| Dr Emrah | Egemen | Pamukkale University, Denizli, Turkey |
| Prof Dr Karl | Rössler | Chair department of neurosurgery, Medical University of Vienna, Austria |
| Prof Thomas | Freiman | Professor and Director, Department of Neurosurgery, University Medical Center Rostock, Germany |
| Dr Malte | Ottenhausen | Head of paediatric neurosurgery, Department of neurosurgery, University Medical Centre Mainz, Germany |
| Dr Verena | Fassl | Pediatric Neurousrgical Department of University Medical Centre of Mainz, Mainz, Germany |
| Prof Bart | Depreitere | Professor of Neurosurgery, Department of Neurosurgery, University Hospitals Leuven, Belgium |
| Dr Marianne | Juhler | Department of Neurosurgery, Copenhagen University Hospital and Department of Clinical Medicine, Copenhagen University, Denmark |
| Dr Gorm | von Oettingen | Head of department, Department of Neurosurgery, Region Midt, Denmark |
| Prof Sandrine | de Ribaupierre | Professor in Department of Clinical Neurological Sciences, Schulich School of Medicine and Dentistry, University of Western Ontario, London, Ontario, Canada |
| Dr Jay | Riva-Cambrin | MD, MSC, Department of Clinical Neurosciences, University of Calgary, Calgary, Canada |
| Dr Adrianna | Ranger | Associate Professor, Department of Clinical Neurological Sciences, Division of Neurosurgery, Schulich School of Medicine and Dentistry, Western University, London, Ontario, Canada |
| Dr Isaac Sunday | Chukwu | Paediatric surgery unit, Federal Medical Centre, Umuahia, Abia State, Nigeria |
| Dr Nikolaos | Syrmos | Aristotle University of Thessaloniki, Greece |
| Dr Aminul | Islam | Pediatric Neurosurgery department,Bangladesh Shishu (Children) Hospital, Dhaka, Bangladesh |
| Prof Jehuda | Soleman | Department of Pediatric Neurosurgery, University Children's Hospital Basel, Switzerland |
| Mr Thangaraj | Munusamy | Division of Neurosurgery, Faculty of Medicine, University of Malaya, Kuala Lumpur, Malaysia; Global Health Research Group in Acquired Brain and Spine Injuries, Cambridge, UK. |
| Prof Dr Vairavan | Narayanan | Division of neurosurgery, Universiti Malaya |
| Dr Gbètoho Fortuné | Gankpe | Dossi Ahouandjinou RCCN and CNHU Hubert Maga, Cotonou, Benin |
| Mr Javier | Ibáñez | Head of neurosurgical department, Son Espases University Hospital, Palma de Mallorca, Spain |
| Mr Conor | Malluci | Department of paediatric neurosurgery, Alder Hey Children's Hospital Trust, Liverpool, UK |
| Dr Shweta | Kedia | MCh Neurosurgery, AIIMs, New Delhi, India |
| Mr William | Lo | Birmingham Children's Hospital, Birmingham, UK |
| Flavio | Giordano | Meyer Children's Hospital - IRCCS University of Firenze, Florence, Italy |
| Prof Suchanda | Bhattacharjee | Nizam's Institute of Medical Sciences, Telangana, India |
| Laurie L | Ackerman | Riley Hospital for Children at Indiana University Health |
| Michael H. | Handler | Children's Hospital Colorado and University of Colorado |
| William | Owen | Neurosurgery, Oxford University Hospitals |
| Ninad | Sawant | Fellow in Pediatric Neurosurgery |
| Mahmoud | Messerer | Unity of Pediatric Neurosurgery, University Hospital Of Lausanne, Centre Hopitalier universitaire vaudois |
| Martina | Messing-Jünger | Asklepios Children´s Hospital, Sankt Augustin, Germany |
| Jose | Hinojosa | Hospital Sant Joan de Déu (Barcelona. Spain) |
| Valentina | Baro | Pediatric and Functional Neurosurgery, Dept. Of Neuroscience, University of Padova |
| Amets | Sagarribay | Hospital Dona Estefânia. Centro Hospitalar Universitário Lisboa Central |
| Valentina | Baro | Pediatric and Functional Neurosurgery, Depr of Neuroscience, University of Padova, Padova, Italy |
| Marwan | Najjar | American University of Beirut, Beirut, Lebanon |
| Gianpiero | Tamburrini | Fondazione Policlinico Gemelli, IRCCS, Rome, Italy |
| Christian | Auer | 1Johannes Kepler University Linz, Kepler University Hospital, Clinical Institute/Department of Neurosurgery, Altenberger Strasse. 69, 4040 Linz and Wagner-Jauregg Weg 15, 4020 Linz, Austria |
| Carlo | Giussani | Fondazione IRCCS San Gerardo dei Tintori di Monza, University of Milano-Bicocca, School of Medicine, Milan, Italy |
| Mandera | Marek | Department of Peadiatric Neurosurhery, Medical University of SIlesia, Katowice, Poland |
| Antonino | Germanò | University of Messina, Messina, italy |
| Giuseppe | Talamonti | Dep. of Neurosurgery, ASST Niguarda, Milano, Italy |
| Sara | Iglesias | Hospital regional universitario de Malaga, Malaga, Spain |
| Ruth Anne | Mitchell | Sydney Children’s Hospital, Sydney, Australia |
| H.H.K. | Delye | Radboudumc Nijmegen, the Netherlands |
| Laura Grazia | Valentini | Fondazione IRCCS Istituto Neurologico Carlo Besta, Milan, Italy |
| Matthieu | Vinchon | Hospices Civils de Lyon, Lyon, France |
| Bart | Depreitere | Neurosurgery, University Hospitals Leuven, Belgium |
| John | Goodden | Leeds Teaching Hospitals NHS Trust, Leeds, UK |
